# Lack of involvement of lipocortin 1 in dexamethasone suppression of IL-1 release

**DOI:** 10.1155/S0962935193000067

**Published:** 1993

**Authors:** E. F. Morand, D. Rickard, N. J. Goulding

**Affiliations:** 1 Bath Institute for Rheumatic Diseases Trim Bridge BA1 1HD Bath UK; 2 Rheumatology Unit Monash Medical Centre Locked Bag No. 29 Clayton 3168 Australia

## Abstract

The annexin lipocortin 1 is reported to mediate some anti-inflammatory effects of glucocorticoids, but the mechanisms of this mediation are incompletely understood. The involvement of lipocortin 1 in glucocorticoid inhibition of monocyte interleukin 1β (IL-1β) release has been investigated. Treatment of peripheral blood monocytes with 2 μg/ml lipopolysaccharide potently increased IL–1β release (p = 0.001) and dexamethasone (10^−7^ M) significantly reduced both resting and stimulated IL-1β release (p = 0.009). A neutralizing monoclonal antibody to lipocortin 1 (0.5–50.0 μg/ml) was unable to inhibit this effect and recombinant lipocortin 1 (2 × 10^−6^ M) and 188aa lipocortin 1 fragment (10^−8^−10^−6^ M) had no effect. It is concluded that lipocortin 1 is not involved in the inhibition of monocyte IL-1β release by glucocorticoids.


Mediators of Inflammation, 2, 49–52 (1993)

### Lack of involvement of lipocortin 1 in dexamethasone suppression of IL-1 release

E. F. Morand[CA,*], D. Rickard and N. J. Goulding

Bath Institute for Rheumatic Diseases, Trim Bridge, Bath, BA1 1HD, UK

* *Present Address:* Rheumatology Unit, Monash Medical Centre, Locked Bag No. 29, Clayton, 3168, Australia

[CA] Corresponding Author

**The annexin lipocortin 1 is reported to mediate some anti-inflammatory effects of glucocorticoids, but the mechanisms of this mediation are incompletely understood. The involvement of lipocortin 1 in glucocorticoid inhibition of monocyte interleukin 1β (IL-1β) release has been investigated. Treatment of peripheral blood monocytes with 2 μg/ml lipopolysaccharide potently increased IL-1β release ($p = 0.001$) and dexamethasone ($10^{-7}$ M) significantly reduced both resting and stimulated IL-1β release ($p = 0.009$). A neutralizing monoclonal antibody to lipocortin 1 (0.5–50.0 μg/ml) was unable to inhibit this effect and recombinant lipocortin 1 ($2 \times 10^{-6}$ M) and 188aa lipocortin 1 fragment ($10^{-8}$–$10^{-6}$ M) had no effect. It is concluded that lipocortin 1 is not involved in the inhibition of monocyte IL-1β release by glucocorticoids.**

**Key words:** Glucocorticoid, Interleukin 1, Lipocortin 1 (annexin 1), Monocyte

### Introduction

Lipocortin 1 (annexin 1) is a member of the annexin family of calcium–phospholipid binding proteins.[1,2] The production of lipocortin 1 is induced by glucocorticoids in a number of systems,[3–6] including human peripheral blood mononuclear cells after *in vivo* exposure to glucocorticoids.[7] Lipocortin 1 has been demonstrated to have a number of anti-inflammatory actions in both *in vitro* and *in vivo* systems,[8–16] but the influence of this protein on cytokine production is unknown. The anti-inflammatory activity of lipocortin 1 *in vivo* has yet to be fully explained in terms of specific actions.

Interleukin-1 (IL-1) is a potent pro-inflammatory cytokine which is produced in a wide range of tissues including tissue macrophages, peripheral blood monocytes, brain, synovium, lung, gut, and bone.[17] It is involved in the mediation of inflammation in a diverse list of conditions including rheumatoid arthritis.[18,19] The production of IL-1 in inflammatory tissue sites is under the control of regulatory and counter-regulatory systems. The major inhibitors of IL-1 production are the glucocorticoids, and it is now well established that dexamethasone inhibits the induction of monocyte IL-1 release by bacterial lipopolysaccharide (LPS) in a dose dependent fashion.[20] The mechanisms of this inhibition are complex and include translational, transcriptional and post-transcriptional events.[21–23] An attractive explanation for some of the anti-inflammatory actions of lipocortin 1 would be the inhibition of IL-1 release or activity, and the possibility that lipocortin 1 is involved in the suppression by glucocorticoids of IL-1β release is supported by several observations. First, glucocorticoid inhibition of IL-1 release in some *in vitro* settings is abrogated by cycloheximide, an inhibitor of protein synthesis.[24] Secondly, nuclear run-off studies suggest that glucocorticoid inhibition of the early phases of monocyte IL-1β release may occur without effects on transcription of the IL-1β gene.[22] The mechanism of transport of IL-1β from the cytoplasm to the extracellular environment is not known, but IL-1β does not appear to have a signal peptide and is not transported via the Golgi apparatus.[25] Annexins, often cytoskeletal associated, have been reported in preliminary studies to be implicated in cell membrane vesicle formation, exocytosis, and secretion.[26,27]

The role of lipocortin 1 in the inhibition by dexamethasone of IL-1β release from peripheral blood monocytes has been investigated using recombinant lipocortin 1, a bioactive lipocortin 1 fragment, and a neutralizing antibody to lipocortin 1. It is reported that none of these agents impact on LPS induced monocyte IL-1β release, or the suppression of it by glucocorticoids, and the authors conclude that lipocortin 1 is not involved in this action of glucocorticoids.

### Materials and Methods

*Reagents:* Cells were cultured in RPMI 1640 (Gibco, UK) supplemented with penicillin, streptomycin and L-glutamine (Gibco, UK) and with 10% heat inactivated charcoal stripped foetal calf serum (Flow, ICN Laboratories, UK). Cell washes were performed with calcium and magnesium-free

© 1993 Rapid Communications of Oxford Ltd phosphate buffered saline with 0.16% glucose (PBSG). Refolded recombinant human lipocortin 1 (rhLC1) and a neutralizing mouse monoclonal antibody to human LC1 (1A) were kindly provided by Dr J. Browning (Biogen, Cambridge, MA). A bioactive N-terminal 188 amino acid fragment of lipocortin 1 (1-188aa) was kindly provided by Dr F. Carey, ICI Pharmaceuticals, Cheshire, UK. IL-1$\beta$ ELISA were purchased from Cascade Biochem (Reading, UK). Dexamethasone and LPS (*Escherichia coli*, serotype 055:B5 lipopolysaccharide) were purchased from Sigma (St Louis, MO).

*Monocyte separation:* Peripheral venous blood was drawn from healthy volunteers into heparinized containers and diluted 1:1 with PBSG. Mononuclear cells were separated by centrifugation on a Histopaque 1077 (Sigma, St Louis, MO) density gradient for 30 min at 400 × *g*, washed in PBSG, and resuspended at $5 \times 10^6$ cells/ml in culture medium with 10% FCS. Monocytes in this suspension were allowed to adhere to 10 cm Petri dishes (Costar, Cambridge, MA) for 60 min at 37°C and 5% $CO_2$ in a humidified incubator. After non-adherent cells were removed by vigorous pipetting with medium, adherent cells were removed by gentle scraping with a rubber 'policeman' and washing with cold PBSG. Adherent cells were <10% CD3 positive by flow cytometric analysis.

*Cell culture:* Monocytes were cultured in $1 \times 10^6$ cell aliquots. Neutralizing antibody to lipocortin 1 (0.5–50 $\mu$g/ml), control antibody P3 (50 $\mu$g/ml), rhLC1 ($2 \times 10^{-6}$ M) or 1-188aa fragment ($2 \times 10^{-6}$ to $2 \times 10^{-8}$ M) were incubated with monocytes for 2 h in 96-well plates at 37°C and 5% $CO_2$ in a humidified incubator. Cells were then resuspended into 1 ml of medium in 24-well tissue culture plates (Costar, Cambridge, MA) and LPS 2 $\mu$g/ml and/or dexamethasone $10^{-7}$ M added. Cells were cultured for 48 h at 37°C and 5% $CO_2$ in a humidified incubator and viability at this time was >95%.

*IL-1$\beta$ assay:* Culture supernatants were obtained by centrifuging plates at 400 × *g* for 5 min and careful aspiration. Supernatants contained $<1 \times 10^4$ cells/ml. Supernatants were stored at −70°C until assay. IL-1$\beta$ ELISA were performed according to the manufacturer's instructions and had a sensitivity of 1 pg/ml.

*Statistical analysis:* Supernatant IL-1$\beta$ levels were compared using the Wilcoxon signed ranks test, or Mann Whitney *U* test when the number of pairs was less than six. Values of $p$ less than 0.05 were regarded as statistically significant.

### Results

IL-1$\beta$was detected in the supernatants of untreated monocytes (mean 623, S.E.M. 122 pg/ml, $n = 13$). In all experiments, LPS 2 $\mu$g/ml induced significant increases in supernatant IL-1$\beta$ concentration (mean 2188, S.E.M. 298 pg/ml, $p = 0.001$, $n = 13$). Dexamethasone potently inhibited LPS induced IL-1$\beta$ release in all experiments (mean 666, S.E.M. 94 pg/ml, $p = 0.009$, LPS *vs* LPS plus dexamethasone, $n = 13$) (Figs 1–3). Dexamethasone $10^{-7}$ M also inhibited the levels of IL-1$\beta$ in the supernatants of non-LPS treated monocytes (mean 291, S.E.M. 85 pg/ml, $p = 0.009$, dexamethasone treated *vs* untreated, $n = 7$, Fig. 1 and 2). Pretreatment of monocytes with neutralizing antibody to lipocortin 1 in doses of 0.5–50.0 $\mu$g/ml had no effect on the inhibitory action of dexamethasone $10^{-7}$ M on IL-1$\beta$ release (Fig. 1). Pretreatment of monocytes with rhLC1 $2 \times 10^{-6}$ M had no suppressive effect on non-LPS treated monocyte IL-1$\beta$ release, nor on the increase in IL-1$\beta$ release induced by LPS (Fig. 2). Pretreatment

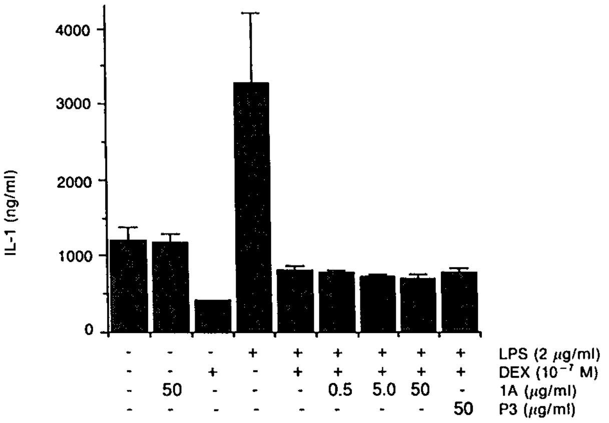

FIG. 1. Monocyte IL-1$\beta$ release: effects of neutralizing antibody to lipocortin 1. Peripheral blood monocytes were cultured for 48 h in the presence of bacterial lipopolysaccharide 2 $\mu$g/ml (LPS), dexamethasone $10^{-7}$ M (DEX), anti-lipocortin 1 antibody 0.5–50 $\mu$g/ml (1A) and/or control antibody (P3), and the IL-1$\beta$ concentration in culture supernatants measured by ELISA. LPS induced a marked increase in IL-1$\beta$ release ($p = 0.001$) which was suppressed by dexamethasone ($p = 0.009$). Dexamethasone also inhibited resting (non-LPS-treated) monocyte IL-1$\beta$ release ($p = 0.009$). Anti-lipocortin antibody had no effect on the ability of DEX to suppress this response.

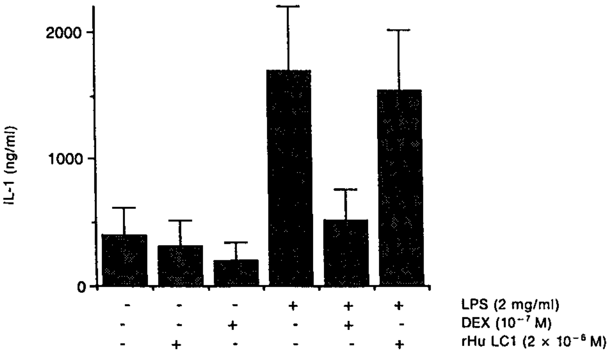

FIG. 2. Monocyte IL-1$\beta$ release: effects of recombinant human lipocortin 1. Peripheral blood monocytes were cultured for 48 h in the presence of LPS 2 $\mu$g/ml, dexamethasone $10^{-7}$ M (DEX), and recombinant human lipocortin 1 $2 \times 10^{-6}$ M (rHLC-1) and the IL-1$\beta$ concentration in culture supernatants measured by ELISA. rhLC-1 did not reproduce the inhibition of monocyte IL-1$\beta$ release observed with dexamethasone.

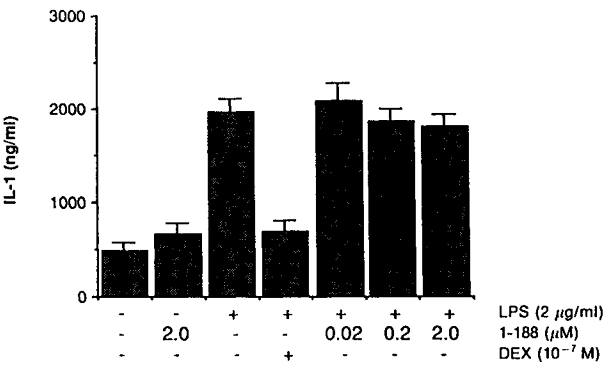

FIG. 3. Monocyte IL-1$\beta$ release: effects of 188aa N-terminal fragment of lipocortin 1. Peripheral blood monocytes were cultured for 48 h in the presence of LPS 2 μg/ml, dexamethasone $10^{-7}$ M (DEX), and N-terminal 188 amino acid lipocortin 1 fragment $2\times10^{-8}$–$2\times10^{-6}$ M (1–188), and the IL-1$\beta$ concentration in culture supernatants measured by ELISA. 1–188 did not reproduce the inhibition of monocyte IL-1$\beta$ release observed with dexamethasone.

of monocytes with the 1-188aa lipocortin 1 fragment at concentrations of $2\times10^{-8}$ M to $2\times10^{-6}$ M similarly had no effect on untreated or LPS treated monocyte IL-1$\beta$ release (Fig. 3).

### Discussion

Evidence from animal models suggests that lipocortin 1, a member of the annexin family of calcium–phospholipid binding proteins, may be a mediator of some of the anti-inflammatory actions of glucocorticoids.[1,2] The production of lipocortin 1 has been shown to be induced by glucocorticoids in a number of *in vitro* and *in vivo* studies.[3–7] Additionally, lipocortin 1 has been demonstrated to mimic many *in vitro* actions of glucocorticoids, including inhibition of natural killer cell activity and antibody dependent cell-mediated cytotoxicity, inhibition of reaction oxygen species generation, and inhibition of prostaglandin and thromboxane release.[8–10] Exogenous lipocortin 1 and bioactive fragments of lipocortin 1 have, furthermore, been demonstrated to exert anti-inflammatory activity *in vivo* in a number of animal models of inflammation.[11–16]

The results reported in this paper do not support a role for lipocortin 1 in the suppression of IL-1$\beta$ release by monocytes. Lipocortin 1 may, however, be involved in the mediation of glucocorticoid inhibition of the actions of IL-1, rather than its production or release. A model for this hypothesis exists in the hypothalamo–pituitary–adrenal axis. IL-1 is produced in the pituitary, IL-1 receptors have been demonstrated in pituitary cell cultures, and circulating IL-1 is active in the pituitary where it is involved in the regulation of the hypothalamo–pituitary–adrenal axis response to inflammation.[28–30] Lipocortin 1 has been demonstrated in the rat pituitary,[31] and intracerebroventricular (i.c.v.) infusion of lipocortin 1 or the 1-188aa peptide fragment of lipocortin 1 is associated with a reduction in the pyrogenic response to i.c.v. IL-1,[14] strongly suggesting that lipocortin 1 can directly inhibit actions of IL-1. In addition, IL-1 increases phospholipase A2 (PLA2) activity and leukocyte prostaglandin release,[17,32] while lipocortin 1 reduces the production of prostaglandins via inhibition of PLA2 activity, probably by binding to its substrate.[33] In contrast, prostaglandins have been reported to inhibit the production of IL-1 by monocytes, possibly as part of an autocrine feedback network.[32,34] Potential effects of lipocortin 1 on IL-1 release or action may be reversed by its effect on prostaglandins. These suggestions of an interaction of IL-1 and lipocortin 1 are of course conjectural, and further research on the area of annexin–cytokine interactions is needed.

In summary, lipocortin 1 is a glucocorticoid induced protein whose anti-inflammatory activity remains incompletely understood. A possible mechanism of action of lipocortin 1 is the inhibition of IL-1 release, possibly through effects on its secretion. In studies with recombinant lipocortin 1, a bioactive lipocortin 1 fragment, and neutralizing antibodies to lipocortin 1, the authors have been unable to demonstrate evidence that lipocortin 1 is involved in the suppression by glucocorticoids of the release of IL-1$\beta$ by human peripheral blood monocytes.

### References

1. Peers SH, Flower RJ. The role of lipocortin in corticosteroid actions. *Ann Rev Respir Dis* 1990; **141:** S18–S21.
2. Flower RJ. Lipocortin. *Prog Clin Biol Res* 1990; **349:** 11–25.
3. Ambrose MP, Hunninghake GW. Corticosteroids increase lipocortin 1 in alveolar epithelial cells. *Am J Respir Cell Mol Biol* 1990; **3:** 349–353.
4. Wong WT, Frost SC, Nick HS. Protein-synthesis-dependent induction of annexin I by glucocorticoid. *Biochem J* 1991; **275:** 313–319.
5. Solito E, Raugei G, Melli M, Parente L. Dexamethasone induces the expression of the mRNA of lipocortin 1 and 2 and the release of lipocortin 1 and 5 in differentiated, but not undifferentiated U-937 cells. *FEBS Lett* 1991; **291:** 238–244.
6. Ambrose MP, Hunninghake GW. Corticosteroids increase lipocortin 1 in BAL fluid from normal individuals and patients with lung disease. *J Appl Physiol* 1990; **68:** 1668–1671.
7. Goulding NJ, Godolphin JL, Sharland PR, *et al.* Anti-inflammatory lipocortin 1 production by peripheral blood leucocytes in response to hydrocortisone. *Lancet* 1990; **335:** 1416–1418.
8. Hattori T, Hirata F, Hoffman T, Hizuta A, Herberman RB. Inhibition of human natural killer (NK) activity and antibody dependent cellular cytotoxicity (ADCC) by lipomodulin, a phospholipase inhibitor protein. *J Immunol* 1983; **131:** 662–665.
9. Cirino G, Flower RJ, Browning JL, Sinclair LK, Pepinsky RB. Recombinant human lipocortin 1 inhibits thromboxane release from guinea-pig isolated perfused lung. *Nature* 1987; **328:** 270–272.
10. Maridonneau-Parini I, Errasfa M, Russo-Marie F. Inhibition of $O_2^-$ generation by dexamethasone is mimicked by lipocortin I in alveolar macrophages. *J Clin Invest* 1989; **83:** 1936–1940.
11. Errasfa M, Russo-Marie F. A purified lipocortin shares the anti-inflammatory effect of glucocorticosteroids *in vivo* in mice. *Br J Pharmacol* 1989; **97:** 1051–1058.
12. Errasfa M, Rothhut B, Russo-Marie F. Phospholipase A2 inhibitory activity in thymocytes of dexamethasone-treated mice—Possible implication of lipocortins. *Biochem Biophys Res Commun* 1989; **159:** 53–60.
13. Relton JK, Strijbos PJLM, O'Shaughnessy CT, *et al.* Lipocortin-1 is an endogenous inhibitor of ischemic damage in the rat brain. *J Exp Med* 1991; **174:** 305–310.
14. Carey F, Forder R, Edge MD, *et al.* Lipocortin-1 fragment modifies the pyrogenic actions of cytokines in rats. *Am J Physiol* 1990; **259:** R266–R269.

15. Perretti M, Flower RJ. Lipocortin-1 inhibits the neutrophil infiltration elicited by interleukin-1 in the mouse air pouch. *Br J Pharmacol* 1992; (in press).
16. Cirino G, Peers SH, Flower RJ, Browning JL, Pepinsky RB. Human recombinant lipocortin 1 has acute local anti-inflammatory properties in the rat paw edema test. *Proc Natl Acad Sci USA* 1989; **86**: 3428–3432.
17. Dinarello CA. Biology of interleukin 1. *FASEB J* 1988; **2**: 108–115.
18. Arend WP, Dayer J-M. Cytokines and cytokine inhibitors or antagonists in rheumatoid arthritis. *Arthritis Rheum* 1990; **33**: 305–315.
19. Kirkham B. Interleukin-1, immune activation pathways, and different mechanisms in osteoarthritis and rheumatoid arthritis. *Ann Rheum Dis* 1991; **50**: 395–400.
20. Uehara A, Kohda H, Sekiya C, Takasugi Y, Namiki M. Inhibition of interleukin-1 beta release from cultured human peripheral blood mononuclear cells by prednisolone. *Experientia* 1989; **45**: 166–167.
21. Knudsen PJ, Dinarello CA, Strom TB. Glucocorticoids inhibit transcriptional and post-transcriptional expression of interleukin 1 in U937. *J Immunol* 1987; **139**: 4129–4134.
22. Kern JA, Lamb RJ, Reed JC, Daniele RP, Nowell PC. Dexamethasone inhibition of interleukin 1 beta production by human monocytes. Posttranscriptional mechanisms. *J Clin Invest* 1988; **81**: 237–244.
23. Lew W, Oppenheim JJ, Matsushima K. Analysis of the suppression of IL-1$\alpha$ and IL-1$\beta$ production in human peripheral blood mononuclear adherent cells by a glucocorticoid hormone. *J Immunol* 1988; **140**: 1895–1902.
24. Nishida T, Nakai S, Kawakami T, Aihara K, Nishino H, Hirai Y. Dexamethasone regulation of the expression of cytokine mRNAs induced by interleukin-1 in the astrocytoma cell line U373MG. *FEBS J* 1989; **243**: 25–29.
25. Dinarello CA. Interleukin 1 and interleukin 1 antagonists. *Blood* 1991; **77**: 1627–1652.
26. Geisow MJ, Ali SM, Boustead C, Burgoyne RD, Taylor WR, Walker JH. Structures and functions of a supergene family of calcium and phospholipid binding proteins. *Prog Clin Biol Res* 1991; **349**: 111–121.
27. Zaks WJ, Creutz CE. Evaluation of the annexins as potential mediators of membrane fusion in exocytosis. *J Bioenerg Biomembr* 1990; **22**: 97–120.
28. Spangelo BL, MacLeod RM. The role of immunopeptides in the regulation of anterior pituitary hormone release. *TEM* 1990; **2**: 409–410.
29. Webster EL, Tracey DE, De Souza EB. Upregulation of interleukin-1 receptors in mouse AtT-20 pituitary tumour cells following treatment with corticotropin-releasing factor. *Endocrinology* 1991; **129**: 2796–2798.
30. Saperstein A, Brand H, Audhya T, *et al.* Interleukin 1 beta mediates stress-induced immunosuppression via corticotropin-releasing factor. *Endocrinology* 1992; **130**: 152–158.
31. Strijbos PJLM, Tilders FJH, Carey F, Forder R, Rothwell NJ. Localization of immunoreactive lipocortin-1 in the brain and pituitary gland of the rat. Effects of adrenalectomy, dexamethasone and colchicine treatment. *Brain Res* 1991.
32. Hopkins SJ. Cytokines and eicosanoids in rheumatic diseases. *Ann Rheum Dis* 1990; **49**: 207–211.
33. Davidson FF, Dennis EA, Powell M, Glenney JR Jr. Inhibition of phospholipase A2 by "lipocortins" and calpactins. An effect of binding to substrate phospholipids. *J Biol Chem* 1987; **262**: 1698–1705.
34. Knudsen PJ, Dinarello CA, Strom TB. Prostaglandins posttranscriptionally inhibit monocyte expression of interleukin-1 activity by increasing intracellular cyclic adenosine monophosphate. *J Immunol* 1986; **137**: 3189–3194.

ACKNOWLEDGEMENTS. E.F.M. is the recipient of a Michael Mason Fellowship, Arthritis Foundation of Australia. N.J.G. thanks the Arthritis Research Council, UK for support.

**Received 24 November 1992; accepted 1 December 1992**
